# Bibliography

**Published:** 1895-08

**Authors:** 


### Bibliography.

WORLD'S HISTORY AND REVIEW OF DENTISTRY. Edited, compiled, and revised by Herman Lennmalin, D.D.S., from the most reliable and authentic resources available at Chicago, Ill.

The title of this book fails to explain its exact character, for the contents, valuable as they are, are in no sense a history of dentistry throughout the world, neither is the book a review of that subject, but concerns itself principally with the laws enacted to govern dentistry in the various countries and States of the world.

The first part is devoted to dentistry in the United States, and gives the rise and to some extent the progress of this profession from 1766 to 1820. This is, in the main, taken from "The History of Dental and Oral Science," and covers but eight pages.

This is followed by "The Dental Colleges of the United States," when and where organized, the "Dental Journals," "Statistics of the Number of Dentists by States Associations," and "Dental State Laws."

This latter portion, covering the States and Territories from Alabama to Wyoming, is the most valuable collection of the various laws since the lamented Rehfuss attempted to bring them together in his work on "Dental Jurisprudence." The collection seems to have been made with care, but a compilation, such as this, can never be perfect while the everlasting tinkering with dental laws continues in the several States.

From the one hundred and twenty-one pages devoted to these laws, the author carries his readers through those of Canada and its several provinces, Mexico, Central America, South America, and Surinam; the various countries of Europe, Turkey in Europe, Africa, Asia, Turkey in Asia, Australia, Sandwich Islands, New Zealand, etc.

The author is to be congratulated on the completion of a book which, if absolutely correct, will be the most valuable for reference in its special lines that has been recently issued from the dental press. This compilation has been the great want of college instructors, and in fact every one having to do with students of various countries. The author certainly seems to have exhausted every effort to make the book a reliable guide.

It may be procured of the S. S. White Dental Manufacturing Company, Wilmington Dental Manufacturing Company, H. D. Justi & Son, or of the author, Rockford, Ill. The cost is five dollars.

USEFUL HINTS FOR THE BUSY DENTIST. By William H. Steele, D.D.S. Wilmington Dental Manufacturing Company, Philadelphia, 1895.

This is a second edition of a very valuable publication. It was fully noticed in this journal in the first edition, and all that need be said now of this is that the author has improved in the good qualities of that by valuable additions. The author has adopted the safe plan of presenting "to the busy practical dentist the latest and best methods of our most skilled operators and best writers, arranged in such a manner as to be accessible at a moment's notice."

Illustrations have been employed where necessary to explain the text.

The book covers two hundred and ninety-eight pages of valuable matter, and must prove a most serviceable and convenient book of reference.

Transactions of the Midwinter Fair Dental Congress, held in San Francisco, commencing June 11, 1894. San Francisco, 1895.

This comprises a full report of that meeting now passed into history, and has a distinct value for reference upon library shelves.

It contains some valuable papers and some of no great importance. This, however, is by no means peculiarly applicable to this Congress, but applies to all organizations, local and national.

The Proceedings are neatly arranged and are creditable to all concerned.

The labor connected with this Congress, devolving as it did upon the few, must have been enormous, and the devotion that carried it to a successful completion deserves and should receive unstinted praise from the dental profession throughout the country.

A photographic presentation of the members of the Congress covers the opening page. This, while not very clear, is an excellent reproduction of many faces made familiar to dentists everywhere.

Transactions of the Twenty-fifth Annual Session of the Southern Dental Association, August 2 to 6, 1894, Hygeia Hotel, Old Point Comfort, Va. Wilmington Dental Manufacturing Company, Philadelphia, 1895.

Transactions of the American Dental Association at the Thirty-third and Thirty-fourth Annual Sessions, held at Chicago, Ill., August 12, 1893, and Old Point Comfort, Va., August 7, 1894. S. S. White Dental Manufacturing Company, Philadelphia, 1895.

These two meetings, while held at the same place and with slight variation in time, were distinctly separate bodies. The transactions have, therefore, a value to be judged quite independently of association of time and place.

Both meetings were handicapped by atmospheric conditions, Old Point Comfort excelling itself in discomfort from heat. This had a noticeable effect upon the discussions. The papers were of the usual variety.

